# The assessment of myometrium perfusion in patients with uterine fibroid by arterial spin labeling MRI

**DOI:** 10.1186/s40064-016-3596-0

**Published:** 2016-11-03

**Authors:** Nozomi Takahashi, Osamu Yoshino, Osamu Hiraike, Eriko Maeda, Masanobu Nakamura, Masaaki Hori, Miyuki Harada, Kaori Koga, Shigeru Saito, Tomoyuki Fujii, Yutaka Osuga

**Affiliations:** 1 Department of Obstetrics and Gynecology, The University of Tokyo, Tokyo, 113-8655 Japan; 2 Department of Obstetrics and Gynecology, The University of Toyama, Toyama, 930-0194 Japan; 3 Department of Radiology, The University of Tokyo, Tokyo, 113-8655 Japan; 4 Philips Electronics Japan, Tokyo, 108-8507 Japan; 5 Department of Radiology, The University of Juntendo, Tokyo, 113-8421 Japan

**Keywords:** Infertility, Uterine fibroid, Arterial spin labeling, MRI

## Abstract

**Background:**

It has been suggested that an inadequate blood supply caused by uterine fibroids may lead to decreasing fertility. Therefore, a quantitative evaluation of blood flow in the uterus might be a good tool for infertility treatments. For the first step, the ability to perform arterial spin labeling (ASL)-MRI in pelvic organs was examined by measuring blood flow in the uterine muscle layer.

**Results:**

Three normal volunteer women, seven patients with one uterine fibroid and four patients treated with GnRH analogue for uterine fibroids, were enrolled in this study. Perfusion of normal uterine myometrium was examined using non-enhanced ASL-MRI. The region of interest was set in the uterine muscle layer, with a point in the iliopsoas or gluteus muscle. The ASL perfusion index was calculated as (ASL value in uterus—ASL value in iliopsoas/gluteus muscle). The ASL perfusion indexes in the secretory phase of all 3 volunteers were significantly lower than the indexes in the proliferative phases (P < 0.05). In patients with fibroids, all three types of fibroids (subserosal, intramural and submucosal types) were included. In seven patients harboring a single uterine fibroid, the ASL perfusion indexes of myometrium on the fibroid-positive side increased 4.9 fold compared with that of the fibroid-negative side. With GnRH analogue treatment, ASL perfusion in myometrium decreased to 39% on average (P < 0.05).

**Conclusion:**

We utilized the ASL-MRI technique to evaluate perfusion of uterine myometrium. For clinical use, an inadequate blood supply caused by uterine fibroids is known to lead to decreasing fertility. The ASL-MRI technique might be useful to evaluate blood supply as a quantitative measurement of fertility in patients with uterine fibroids.

## Background

In the uterus, adequate blood flow is important to maintain fertility, while vascular abnormalities are associated with clinical symptoms such as abnormal bleeding, implantation failure and gestation discontinuation (Deligdish and Loewenthal 1970; Buttram and Reiter 1981). In fact, it has been suggested that an inadequate blood supply caused by uterine fibroids may lead to decreasing fertility (Ng and Ho 2002). Therefore, a quantitative evaluation of blood flow in the uterus might be a good tool for infertility treatments. To date, ultrasound techniques, i.e. laser Doppler fluxmetry and power Doppler (Gannon et al. 1997; Raine-Fenning et al. 2004a) have been examined to evaluate blood supply, but a standardized method has not been established.

Recently, arterial spin labeling (ASL) techniques have been examined to assess tissue perfusion without contrast media. ASL is a Magnetic Resonance (MR) perfusion method using magnetic-labeled arterial blood water as an endogenous tracer. ASL is thought to be especially useful for evaluating cerebral blood flow (Telischak et al. 2015), but the use of ASL to evaluate blood flow in pelvic organs has not been established. This non-invasive technique might be useful to evaluate blood flow in the uterus to improve the outcome of infertility treatments.

In the present study, as a first step, we used the ASL technique to examine blood flow in the uterine muscle layer in three separate groups: healthy volunteers, patients with uterine fibroids, and patients with fibroids before and after treatment with GnRH analogues (GnRHa).

## Methods

### Subjects

From August 2012 to September 2013, non-enhanced MR perfusion studies using ASL were performed on volunteers and patients with fibroids as part of the routine pre-operative MRI examination at Yaesu Clinic. This study was approved by the institutional reviewed board, and informed consent was obtained from the patients for the examinations.

### Study design

#### MR perfusion protocols

Uterus perfusion measurements were acquired in an axial slice on a 3 T Philips Achieva (Philips Medical Systems, Best, the Netherlands) with 32ch Cardiac Torso coil using a FAIR single shot Turbo spin-echo acquisition scheme. Briefly, labeling pulse was positioned to cover bilateral common iliac arteries. From 1 to 3 s after labeling, signal intensities were measured at uterus. Detail Parameters were as followed; repetition time/echo time 3600/5.9 ms, echo train length 21, matrix 128 × 96, FOV 230 × 230 mm^2^, imaging/tagging slice thickness 10/200 mm. Imaging time for each inversion time was 90 s. The slice which exhibited the maximum area of uterine fibroid was used.

#### Determination of the optimal scanning time of ASL and a region of interest in the pelvis

**Fig. 1:**
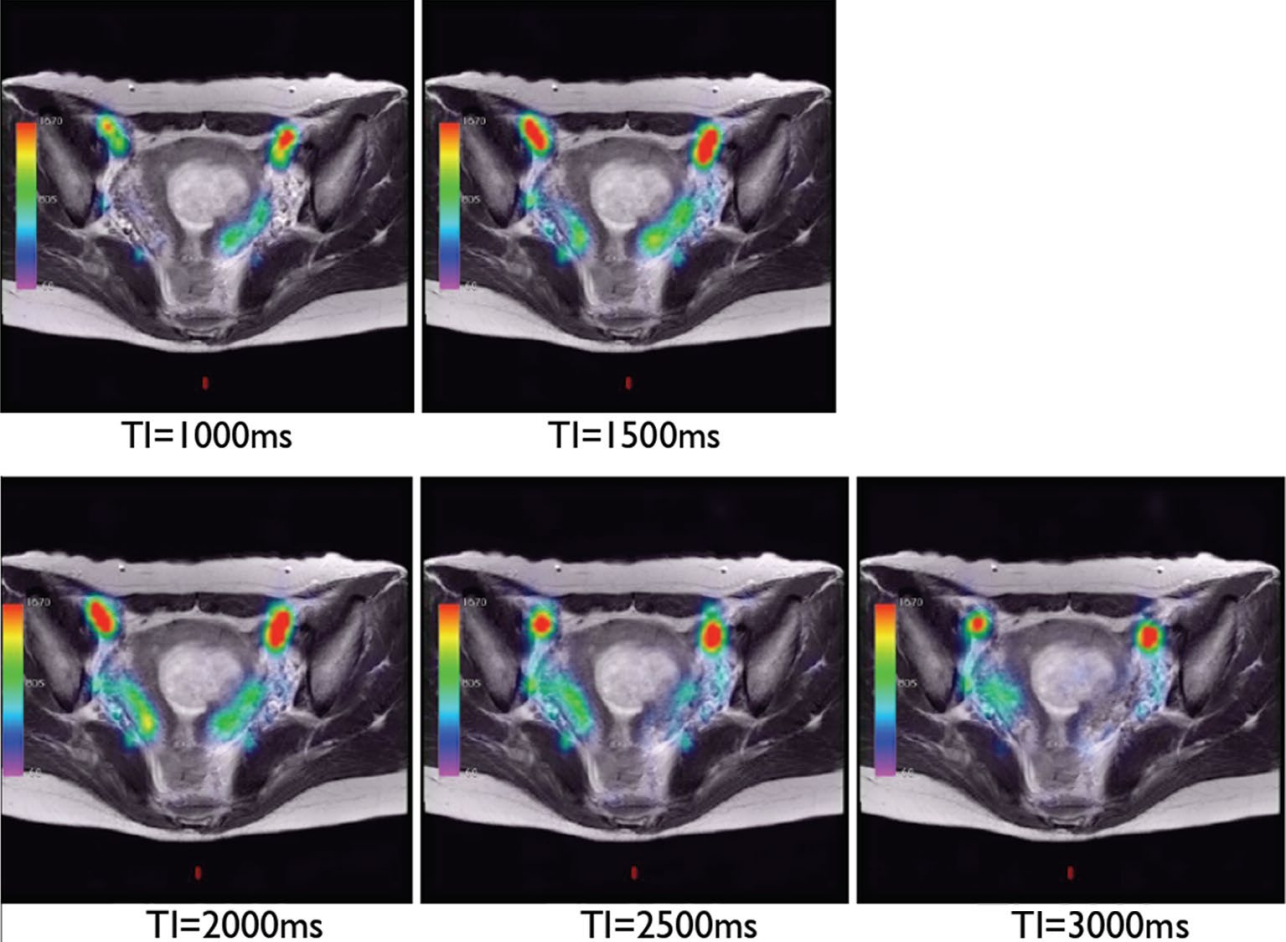
Sequential ASL signals in the uterus. ASL signals in the uterus were observed at 1.0 ms after labeling, and increased until 1.5 or 2.0 ms, then attenuated. We set the optimal scanning time as 1.5 ms after labeling

In a pilot study, we examined various scan timings of the ASL signal after labeling. As shown in Fig. 1, the ASL signals in the uterus were observed at 1.0 s after labeling, and increased until 1.5 or 2.0 s, then attenuated. We set the optimal scanning time as 1.5 s after labeling, which was used for subsequent studies.

A visual evaluation of the colored perfusion map calculated on the MR scanner using software provided by the MR vendor and a region of interest (ROI) analysis were performed by two experienced radiologists. The ROI consisted of a 5 mm^2^ diameter circle. We set 4–5 points of the ROI in the uterine muscle layer, a point in the iliopsoas/gluteus muscle, which was shown in the same image, and a point in the air as background. The perfusion index was calculated as (ASL value in uterus—ASL value in iliopsoas/gluteus muscle). To compare data of different time points, data were corrected by adjusting background ASL value. We examined various subjects as described below.

#### Examination 1) Physiological status of perfusion in the myometrium

To examine the effect of hormonal status, the ASL perfusion indexes of uterine muscle were obtained in proliferative and secretory phase, respectively, from three healthy volunteers who had regular menstrual cycles and normal uterus and ovaries (evaluated via MRI). The uterine cavity was divided into five equal parts in the image, and five points of ROI were set at muscle layer just outside of the junctional zone. ROIs were set at the same locations in both proliferative and secretory phases.

#### Examination 2) Perfusion in the myometrium of patients with fibroids

To examine the effect of uterine fibroids, we evaluated seven patients who each possessed one fibroid. In this examination, all subjects were examined during the proliferative phase. We set 2 ROIs at muscle layer, just outside of the junctional zone, on the fibroid-positive side and -negative side, respectively. We then determined the average ASL perfusion index on each side, and compared the averages for the fibroid-positive and -negative side.

#### Examination 3) Perfusion in myometrium around the fibroid before and after GnRHa treatment

Four patients with uterine fibroids treated with GnRHa were enrolled in this study (one patient with a single fibroid and three patients with multiple fibroids). In this examination, the subjects underwent their first MRI during the proliferative phase, and ROIs were set at myometrium, just outside of the junctional zone. After two or three rounds of GnRHa, the patients underwent a second MRI. ROIs were set at the same locations in myometrium before and after GnRHa treatment, and ASL perfusion indexes were compared.

### Statistical analysis

Statistical analysis was performed by using JMP Pro 11 software (SAS institute Inc., Cary, NC). ASL perfusion indexes are shown as the mean + S.E.M relative to an adjusted value of 1.0 for the mean value of the other one. The data were analyzed by the student’s t test for paired comparison. A P value of less than 0.05 was considered significant.

## Results

### Examination 1) Physiological status of perfusion in myometrium

**Fig. 2:**
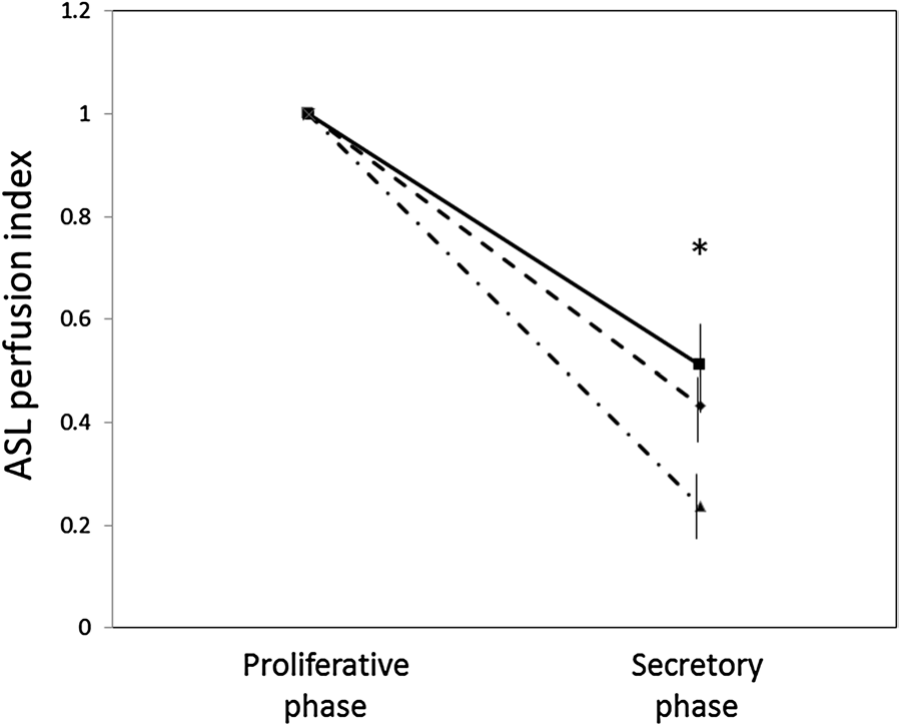
ASL perfusion indexes of normal myometrium in the proliferative and secretory phases. Three healthy volunteers who had normal MRI appearances in the uterus and ovaries with regular menstrual cycles were enrolled in this study. ASL perfusion indexes were calculated as (ASL value in uterus—ASL value in iliopsoas/gluteus muscle) and data were normalized with ASL values in background. Data were shown as the mean + S.E.M relative to an adjusted value of 1.0 for the mean value of the proliferative phase. ASL perfusion indexes in the secretory phase were significantly lower than the levels in the proliferative phase (*P < 0.05)

The ASL perfusion indexes of myometrium in normal uterus were measured in the proliferative and secretory phases. Signal intensities during the secretory phase were evaluated as a relative ratio to that of the proliferative phase. As shown in Fig. 2, the ASL perfusion indexes in the secretory phase of all three volunteers were significantly lower than the indexes in the proliferative phase (P < 0.05).

### Examination 2) The effect of uterine fibroids on perfusion in normal myometrium

**Fig. 3:**
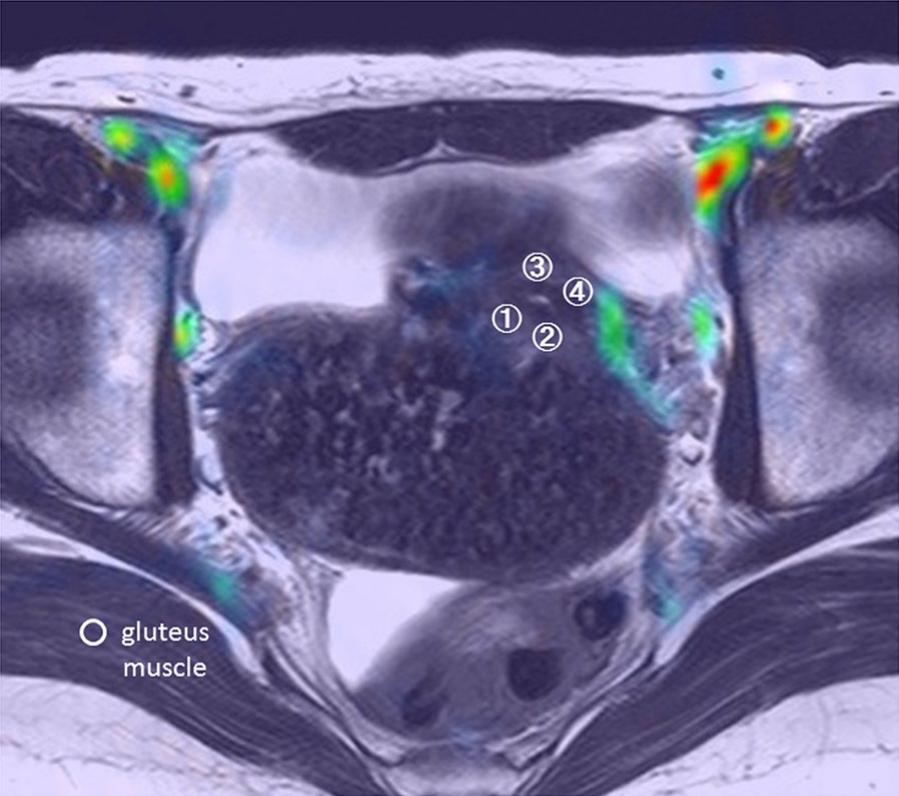
Region of interest of ASL perfusion in a 41-year-old patient with fibroid. The fibroid was subserosal-mural type arising from uterine posterior wall. ROI①: right uterine posterior wall beneath the fibroid, ROI②: left uterine posterior wall beneath the fibroid, ROI③: uterine anterior wall, ROI④: uterine anterior wall, and gluteus muscle. We defined ROI① and ROI② as fibroid positive side, and ROI③ and ROI④ as fibroid negative side. ASL perfusion indexes were calculated as ASL value in uterus—ASL value in gluteus muscle

Figure 3 shows an example of the ROI in this examination. ROIs were set at uterine muscle layer just outside of the junctional zone. This patient had an intramural fibroid arising from the uterine posterior wall. We set 5 points of ROI (ROI①: uterine right posterior wall beneath the fibroid, ROI②: left uterine posterior wall beneath the fibroid, ROI③: right uterine anterior wall, ROI④: left uterine anterior wall and gluteus muscle). We defined ROI① and ② as the fibroid-positive side, and ROI③ and ④ as the fibroid-negative side. As shown in Table 1, the ASL data on the fibroid-positive side were comparable (56.6 and 56.7), and those taken on the fibroid negative side were 2.7 and 8.7. ASL perfusion indexes on the fibroid-positive side were around 9.9 times higher than those measured on the fibroid-negative side.

**Table 1:** ASL values in a 41-year-old patient with fibroid

	ROI①	ROI②	ROl③	ROI④	Gluteus muscle
ASL measured value	707.9	708	654	660	651.3
ASL perfusion indexes (ROI-muscle)	56.6	56.7	2.7	8.7	

ASL perfusion indexes were calculated as ASL value in uterus—ASL value in gluteus muscle

**Fig. 4:**
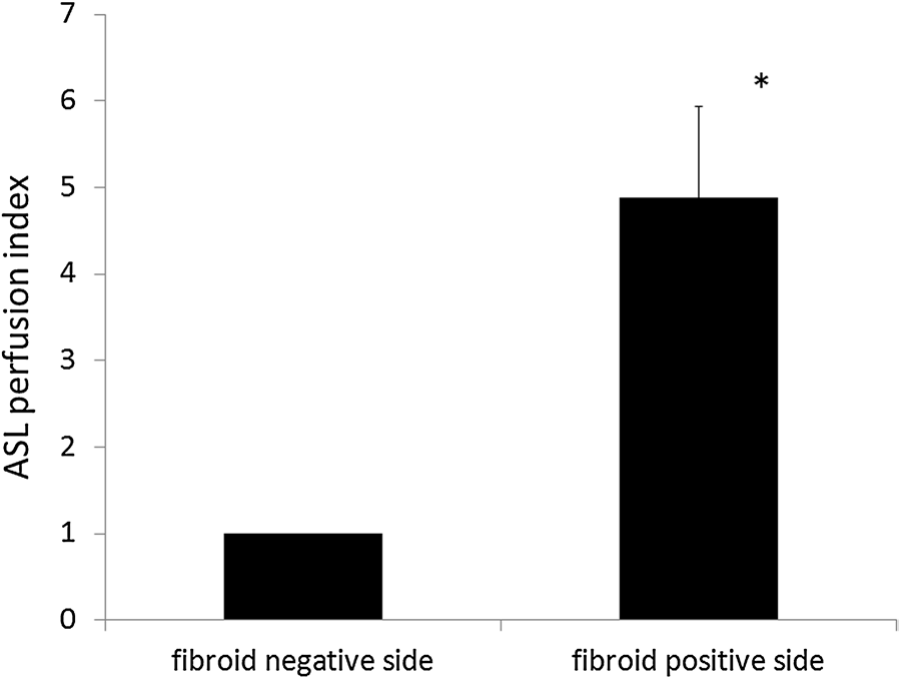
Comparison of ASL perfusion indexes on the fibroid-positive or -negative side. ASL perfusion indexes were calculated as ASL value in uterus—ASL value in iliopsoas/gluteus muscle. Data are shown as the mean ± S.E.M relative to an adjusted value of 1.0 for the mean value of the fibroid negative side. Perfusion of the fibroid-positive side was significantly higher than that of the fibroid-negative side (*P < 0.05)

Table 2 shows the summary of patients’ background and ASL values. All three types of fibroids (subserosal, intramural and submucosal type) were included. Higher ASL perfusion indexes (mean value 4.9 fold increases) were demonstrated on the fibroid-positive side than on the fibroid-negative side (Fig. 4).

**Table 2:** Characteristics and ASL perfusion indexes of patients with uterine fibroids

	Age	Size of uterine fibroid (mm)	Type of fibroid	ASL perfusion inc (ROI-muscle)		
				Fibroid (+)	Fibroid (−)	(+)/(−) Fold
Case 1	41	106 × 56	Subserosal	56.7	5.7	9.9
Case 2	39	68 × 59	Intramural	62.4	7.1	8.6
Case 3	37	68 × 51	Intramural	13.9	1.7	7.8
Case 4	42	70 × 65	Subserosal	175	96.4	1.8
Case 5	40	44 × 36	Intramural	21.1	12.2	1.7
Case 6	38	35 × 28	Submucosal	23.4	4.9	4.6
Case 7	47	72 × 52	Intramural	199	127	1.5

Patients who possessed one fibroid underwent MRI during the proliferative phase. Two ROIs of myometrium on the fibroid-positive side and fibroid-negative side were set. ASL perfusion indexes were calculated as ASL value in uterus—ASL value in iliopsoas/gluteus muscle

### Examination 3) The effect of GnRHa treatment on perfusion in myometrium around fibroids

The effect of GnRHa on ASL perfusion in myometrium was evaluated. ASL data obtained from 4 ROIs were averaged. The fibroid size and ASL perfusion index of all patients decreased after GnRHa treatment (Table 3). The percentage change in the ASL perfusion index ranged from 6.8 to 73%, giving a decrease to 39% of basal levels on average (P < 0.05).

**Table 3:** Characteristics and ASL perfusion indexes of patients with fibroids treated with GnRH analogue

	Age	Diameter of fibroid (mm) Before ⇒ after GnRH treatment (reduction ratio)	Type of fibroid	Calibrated ASL perfusion index
				(%) After ⇒ before
Case 1	39	86 ⇒ 80 (7%) 72 ⇒ 72 (0%) 56 ⇒ 42 (25%) 52 ⇒ 52 (0%)	Subserosal Subserosal Subserosal Intramural	15% (8.0 ⇒ 1.2)
Case 2	39	64 ⇒ 64 (0%) 40 ⇒ 25 (38%)	Intramural intramural	63% (9.9 ⇒ 6.3)
Case 3	44	18 ⇒ 16 (11%}	Submucosal	6.8% (12.9 ⇒ 0.88)
Case 4	45	96 ⇒ 70 (27%) 42 ⇒ 30 (29%)	Intramural Intramural	73% (11.7 ⇒ 8.6)
				Mean 39%

The subjects underwent their first MRI during the proliferative phase. After two or three rounds of GnRH analogue administration, the patients underwent a second MRI. ROIs were set at the same locations in myometrium beneath fibroids. ASL perfusion indexes before and after GnRH analogue treatments were calculated as (ASL value in uterus—ASL value in iliopsoas/gluteus muscle). Data were calibrated with ASL values in background

There is no correlative relationship between the decreasing ratio of fibroid size and change in ASL index before and after GnRHa.

## Discussion

In the present study, we utilized the ASL-MRI technique to evaluate perfusion of myometrium, and showed the ability to perform the technique by the findings that (1) normal myometrium perfusion during the secretory phase was significantly lower than that of the proliferative phase, (2) perfusion of myometrium on the fibroid-positive side was significantly higher than that of the fibroid-negative side and (3) with GnRHa treatment, ASL perfusion in myometrium decreased to 39% on average.

The two most common methods for measuring perfusion with MRI are based on dynamic susceptibility contrast (DSC) and arterial spin labeling (ASL) (Wolf and Detre 2007). As MR perfusion using ASL does not need contrast medium, and ASL signals disappear in few seconds, it is a non-invasive examination and can be performed repeatedly even in renal failure patients (Telischak et al. 2015). ASL MRI is useful to diagnose various cerebrovascular diseases which cause a disorder of perfusion (Detre et al. 2012), and it has also been used to examine normal brain function (Wang et al. 2003; Telischak et al. 2015). We applied ASL-MRI to the female pelvis in order to evaluate perfusion in the myometrium. In our study, ASL perfusion in normal myometrium during the secretory phase was lower than in the proliferative phase. In accordance with our findings, ultrasound techniques designed to measure blood flow also revealed that myometrium showed lower tissue blood flow during the secretory phase compared to the proliferative phase by laser Doppler fluxmetry and power Doppler angiography (Gannon et al. 1997; Raine-Fenning et al. 2004a). During the proliferative phase, small vessels, including the spiral and straight arterioles in the inner myometrium develop (Blackwell and Fraser 1988). Additionally, the increase of serum estradiol levels and the decrease in microvascular resistance during the proliferative phase are known to increase blood flow (de Vries et al. 1990).

Secondly, we used the ASL-MRI technique to demonstrate the influence of fibroids in myometrium perfusion. Doppler ultrasound examination revealed that in women with fibroids, blood flow in the uterine artery was higher than in women without fibroids (Sladkevicius et al. 1996; Kurjak et al. 1992; Alataş et al. 1997). However, it is difficult to evaluate perfusion of local myometrium. Using the ASL technique, we were able to evaluate perfusion in local regions by setting the region of interest (ROI). In our study, we evaluated the perfusion of myometrium beneath the endometrium, and found that all three types, submucosal, intramural, and subserosal fibroids, increased blood perfusion in the adjacent myometrium. Importantly, the ASL signal in the myometrium on the fibroid-positive side was higher than that of fibroid-negative side, implying that fibroids may cause an imbalance of blood distribution in the myometrium.

In recent studies, fibroids have been shown to exhibit high levels of pro-angiogenic factors, which cause an increase in angiogenesis and vascular density in the normal myometrium surrounding fibroids (Tal and Segars 2014). Vascular abnormalities due to the presence of fibroids might be associated with clinical symptoms such as abnormal bleeding, implantation failure and gestation discontinuation (Deligdish and Loewenthal 1970; Buttram and Reiter 1981). In another report, endometrial and sub-endometrial perfusion was impaired in women with unexplained subfertility (Raine-Fenning et al. 2004b). These data imply that an adequate blood supply to the endometrium is required for implantation. In some patients with fibroids, one of the symptoms is infertility. Meta-analyses showed that submucosal but not subserosal fibroids have a negative effect on pregnancy rate, while conclusions regarding intramural lesions have been conflicting (Somigliana et al. 2007; Pritts et al. 2009). Therefore, subserosal and intramural fibroids are not routinely removed for infertility treatment. However, in some patients, removal of fibroids could improve pregnancy rate and decrease miscarriage (Brady et al. 2013). Gynecologists need to accurately assess the effectiveness of surgical intervention of uterine fibroids. In our study, although it included a small numbers of patients, all three types of fibroids caused an imbalance of perfusion in uterine myometrium. Moreover, there was no correlation between ASL perfusion indexes and types or the size of fibroid. Further study is needed to investigate the relationship between uterine perfusion and reproductive outcomes.

Finally, we confirmed that the ASL-MRI technique could detect the decrease of myometrial blood supply with GnRHa treatment. The use of GnRHa prior to fibroid surgery reduces fibroid volume, and also improves pre-operative anemia, and intra-operative blood loss (Lethaby et al. 2001). GnRHa treatment led to a decrease in micorovascular density in tissue of uterine fibroids (Khan et al. 2010), and a decrease in angiogenesis-related factors such as VEGF, bFGF, and PDGF (Di Lieto et al. 2005). Consistent with our ASL-MRI finding, there are some reports that GnRHa treatment reduced the uterine blood flow evaluated with Doppler ultrasound (Matta et al. 1988; Aleem and Predanic 1995). However, in the present study, there is no correlative relationship between the decreasing ratio of fibroid size and change in ASL index before and after GnRHa, suggesting that other factors might be involved in decreasing fibroid size.

In the present study, we could not compare ASL signal data with perfusion data obtained by using contrast agents, due to the clinical limitations in which contrast agents were not used for patients with fibroids in our clinic. In the brain MRI, it appears that blood flow can be estimated correctly in the grey matter of the brain by using ASL techniques (Wintermark et al. 2005). Further study is needed to determine whether ASL signal actually correlates to the blood flow in the uterus.

## Conclusion

We evaluated myometrium perfusion using ASL-MRI. This technique has successfully been performed in the uterus to assess tissue perfusion without contrast media. Future uses of this technique may include fibroid analysis in the investigation into infertility and assessment of malignant tumors.
